# Comparative Molecular Docking of Apigenin and Luteolin Versus Conventional Ligands for TP-53, pRb, APOBEC3H, and HPV-16 E6: Potential Clinical Applications in Preventing Gynecological Malignancies

**DOI:** 10.3390/cimb46100661

**Published:** 2024-10-03

**Authors:** Momir Dunjic, Stefano Turini, Lazar Nejkovic, Nenad Sulovic, Sasa Cvetkovic, Marija Dunjic, Katarina Dunjic, Dina Dolovac

**Affiliations:** 1 School of Medicine, University of Pristina, BB Anri Dinana, 38220 Kosovska Mitrovica, Serbia; sulovicnenad@yahoo.com; 2 Faculty of Pharmacy, Heroja Pinkija 4, 21000 Novi Sad, Serbia; 3 Alma Mater Europaea (AMEU-ECM), Slovenska Ulica/Street 17, 2000 Maribor, Slovenia; turini.stefano@yahoo.it; 4 BDORT Center for Functional Supplementation and Integrative Medicine, Bulevar Oslobodjenja 2, 11000 Belgrade, Serbia; dunjickatarina@gmail.com; 5 Guard Plus Doo, Nemanjina 40, 11000 Belgrade, Serbia; 6 Worldwide Consultancy and Services, Division of Advanced Research and Development, Via Andrea Ferrara 45, 00165 Rome, Italy; marijadunjic@yahoo.com; 7 Capri Campus Forensic and Security, Division of Environmental Medicine and Security, Via G. Orlandi 91 Anacapri, Capri Island, 80071 Naples, Italy; 8 Belgrade University, School of Medicine, dr Subotića Starijeg 8, 11000 Belgrade, Serbia; lazar.nejkovic@gmail.com; 9 Clinic for Obstetrics and Gynecology, Kraljice Natalije 62, 11000 Belgrade, Serbia; 10 General Hospital, UI. Generala Zivkovica 1, 36300 Novi Pazar, Serbia; dinadolovac95@gmail.com

**Keywords:** molecular docking, HPV-16, TP-53, pRb, APOBEC, apigenin, luteolin, protein–protein interaction, high-performance computing (HPC), oncoprotein E6

## Abstract

This study presents a comparative analysis of molecular docking data, focusing on the binding interactions of the natural compounds apigenin and luteolin with the proteins TP-53, pRb, and APOBEC, in comparison to conventional pharmacological ligands. Advanced bioinformatics techniques were employed to evaluate and contrast binding energies, showing that apigenin and luteolin demonstrate significantly higher affinities for TP-53, pRb, and APOBEC, with binding energies of −6.9 kcal/mol and −6.6 kcal/mol, respectively. These values suggest strong potential for therapeutic intervention against HPV-16. Conventional ligands, by comparison, exhibited lower affinities, with energies ranging from −4.5 to −5.5 kcal/mol. Additionally, protein–protein docking simulations were performed to assess the interaction between HPV-16 E6 oncoprotein and tumor suppressors TP-53 and pRb, which revealed high binding energies around −976.7 kcal/mol, indicative of their complex interaction. A conversion formula was applied to translate these protein–protein interaction energies to a comparable scale for non-protein interactions, further underscoring the superior binding potential of apigenin and luteolin. These findings highlight the therapeutic promise of these natural compounds in preventing HPV-16-induced oncogenesis, warranting further experimental validation for clinical applications.

## 1. Introduction

### 1.1. Human Papillomavirus (HPV-16)

Human papillomavirus (HPV) is a non-enveloped DNA virus within the Papillomaviridae family, comprising over 200 genotypes [1]. HPV-16 is one of the most oncogenic strains, significantly contributing to the development of cervical cancer and other anogenital and oropharyngeal malignancies [2]. The HPV-16 genome is a double-stranded circular DNA molecule approximately 8000 base pairs in length [3]. It encodes eight early (E) proteins (E1, E2, E4, E5, E6, E7, E8) essential for viral replication and oncogenesis [4], and two late (L) proteins (L1, L2) involved in virion assembly [5]. E6 and E7 are particularly critical due to their roles in disrupting host cell cycle control mechanisms, specifically targeting the tumor suppressor proteins TP-53 and pRb [6].

HPV-16 replication occurs in the basal epithelial cells, initiated when the virus infects basal keratinocytes through micro-abrasions [7]. The viral DNA enters the nucleus and is maintained as an episome. Early proteins E1 and E2 mediate the replication and transcription of viral DNA [8]. E6 and E7 disrupt cellular regulatory pathways by targeting TP-53 and pRb, promoting cellular proliferation and viral replication [9]. As infected cells differentiate and move towards the epithelial surface, late genes L1 and L2 are expressed [10], leading to the assembly of new virions and the continuation of the infection cycle [11].

HPV-16 is a major etiological agent in cervical cancer [12], responsible for approximately 50% of cases worldwide [13]. The oncogenic potential of HPV-16 is primarily due to the actions of E6 and E7. E6 promotes the degradation of the tumor suppressor TP-53, inhibiting apoptosis and allowing the accumulation of genetic mutations [14]. E7 binds to pRb, releasing E2F transcription factors, leading to uncontrolled cell cycle progression. These interactions result in genomic instability, increased cell proliferation, and eventually malignant transformation [15].

Conventional treatments for HPV-related malignancies include chemotherapeutic agents, like cisplatin, 5-fluorouracil, and radiotherapy [16]. Targeted therapies, such as immune checkpoint inhibitors, are also being explored [17]. Prophylactic vaccines, like Gardasil and Cervarix, have been highly effective in preventing HPV infections by inducing an immune response against the L1 protein [18]. However, therapeutic options for existing infections are limited, highlighting the need for novel therapeutic strategies [19].

### 1.2. Molecular Docking in HPV Research

Molecular docking is a computational technique used to predict the interaction between a ligand and a protein, identifying potential drug candidates by predicting their binding affinity to target proteins [20]. In the context of HPV, molecular docking can help identify natural compounds that might disrupt the interactions between HPV oncoproteins and crucial cellular proteins, like TP-53 and pRb. This study utilizes molecular docking to compare the binding affinities of two conventional pharmacological ligands and natural compounds, namely apigenin and luteolin, with TP-53, pRb, and APOBEC.

### 1.3. Artificial Intelligence (AI) and Its Role in Drug Discovery

Artificial intelligence (AI) has become an integral tool in drug discovery, aiding in the prediction and optimization of molecular interactions [21]. In this study, AI was employed to suggest two potential natural compounds, apigenin and luteolin, based on their structural characteristics and predicted binding affinities to key HPV-related proteins. While the AI’s role was limited to this preliminary identification, it provided valuable insights that guided subsequent molecular docking studies. 

### 1.4. TP-53, pRb, and APOBEC: Targets of HPV Oncoproteins

TP-53 (tumor protein P53), pRb (retinoblastoma protein), and APOBEC (apolipoprotein B mRNA editing enzyme, catalytic polypeptide) are critical regulators of cellular processes, including cell cycle control, apoptosis, and antiviral defense. TP-53, known as the “guardian of the genome”, regulates DNA repair and apoptosis [22]. pRb controls cell cycle progression by inhibiting E2F transcription factors [23]. APOBEC proteins, particularly APOBEC3H, induce hypermutation in viral genomes, contributing to antiviral defense [24]. The HPV-16 oncoproteins E6 and E7 subvert these mechanisms, promoting oncogenesis [25,26]. 

### 1.5. Natural Compounds Apigenin and Luteolin

Apigenin and luteolin are natural flavonoids with demonstrated anticancer and antiviral properties [27]. Their ability to bind to and stabilize tumor suppressor proteins, such as TP-53 and pRb, while potentially disrupting HPV oncoprotein interactions, positions them as promising candidates for therapeutic development. Molecular docking studies indicate that these compounds exhibit significant binding affinities to TP-53, pRb, and APOBEC, suggesting their potential role in preventing HPV-16-related oncogenesis.

## 2. Materials and Methods

### 2.1. Software and Tools

#### 2.1.1. 1-Click Docking

The 1-Click Docking software (https://mcule.com/apps/1-click-docking/, accessed on 28 September 2024) was employed for the initial molecular docking studies between non-protein ligands and protein targets. This tool facilitates rapid and accurate docking simulations, allowing for efficient screening of potential ligands. The software’s algorithm identifies high-affinity binding conformations by evaluating multiple possible binding poses.

#### 2.1.2. ClusPro 2.0

ClusPro 2.0 (https://cluspro.org/login.php?redir=/queue.php, accessed on 28 September 2024), a widely recognized protein–protein docking software, was used to study interactions between protein ligands and protein receptors. This software integrates clustering algorithms to predict the most likely binding conformations based on energy minimization. Protein–protein docking studies utilized a remote supercomputer connection, providing the necessary computational power for handling complex simulations.

#### 2.1.3. ChatGPT-4

ChatGPT-4 was employed to suggest natural molecules with high binding affinity to TP-53 (PDB ID: 8DC4), pRb (PDB ID: 4CRI), and APOBEC3H (PDB ID: 5Z98). The platform facilitated initial screening by proposing apigenin and luteolin as potential candidates based on their chemical and biological properties. 

#### 2.1.4. Role of ChatGPT-4 in Pharmacological-Ethnobotanical Molecule Screening and Training Procedure

In this study, ChatGPT-4 was employed as an advanced tool for identifying natural compounds with high potential for therapeutic applications, particularly in the fields of pharmacology and ethnobotany. A key aspect of this process was the detailed and precise training of the AI model, which involved crafting highly specific queries designed to guide the AI towards identifying molecules with superior binding affinity for molecular targets, such as TP-53, pRb, and APOBEC3H.

#### 2.1.5. Training Procedure and Specific Query Design

The training of ChatGPT-4 for this purpose required a structured and iterative process, where the AI model was gradually refined through a series of increasingly specific questions [28]. These questions were formulated based on critical parameters relevant to pharmacological screening, such as molecular weight thresholds, the presence of bioactive functional groups (e.g., hydroxyl groups, flavonoid structures), and known interactions with viral and oncogenic proteins. For instance, questions were designed to focus the AI’s search on flavonoids with strong antioxidative and antiviral properties, directing it to molecules that are structurally similar to apigenin and luteolin. Queries, such as “Identify plant-derived flavonoids with a molecular weight between 250 and 350 g/mol that exhibit strong interactions with p53 and possess hydroxyl groups at specific positions on the B-ring” were employed. This ensured that the AI targeted compounds with a structural framework known to enhance binding affinity, while excluding irrelevant candidates. To further refine the screening process, follow-up questions were posed to evaluate the pharmacokinetic properties, such as the bioavailability, solubility, and potential metabolic pathways of the suggested compounds. The iterative nature of this process allowed the AI to narrow down its selection to molecules that not only showed strong binding potential but also favorable pharmacological properties. This specific query-based training was instrumental in guiding ChatGPT-4 to identify apigenin and luteolin as leading candidates. The process leveraged the AI’s ability to cross-reference vast amounts of pharmacological and chemical data, effectively synthesizing information across multiple databases and research papers. By asking highly detailed and pointed questions, the research team was able to train ChatGPT-4 to focus on molecules that aligned with the desired pharmacological profiles.

#### 2.1.6. Artificial Intelligence as a Tool in Pharmacological–Ethnobotanical Screening

The application of ChatGPT-4 in this context showcases the power of AI in streamlining the drug discovery process, particularly when working with natural compounds derived from ethnobotanical sources [29] The AI’s ability to rapidly process and analyze large datasets allows researchers to conduct extensive screenings without the traditional bottlenecks of manual data analysis. By integrating highly specific training queries, the AI can be directed to prioritize molecules that meet strict pharmacological criteria, including efficacy, safety, and bioavailability [30].

#### 2.1.7. Implications for Future AI Integration in Ethnobotanical and Pharmacological Research

The success of this approach signals a future where AI models, like ChatGPT-4, will play an increasingly central role in pharmacological and ethnobotanical research. The ability to train AI through tailored questions offers a scalable solution for rapid screening and identification of candidate molecules from vast libraries of natural products. As AI continues to evolve, its utility in identifying compounds with superior binding affinities and therapeutic potential will be invaluable, particularly in areas where natural product diversity offers untapped pharmacological opportunities. The integration of AI models that can be precisely trained through targeted questioning also opens the door to more dynamic applications in drug discovery [31]. By refining the model with domain-specific knowledge, researchers can harness AI to explore complex chemical spaces, optimizing the discovery of novel therapeutic agents that are rooted in traditional ethnobotanical knowledge while leveraging cutting-edge computational tools. This methodology of training AI through specific and detailed queries not only accelerates the drug discovery process but also ensures that the identified compounds meet rigorous scientific standards for future pharmacological development. ChatGPT-4’s performance in this study exemplifies the potential of AI-driven research in bringing novel therapeutic strategies, particularly those derived from plant-based compounds, into mainstream medicine [32].

### 2.2. Workflow

#### 2.2.1. Step 1: Identification of Natural Compounds

ChatGPT-4 was used to identify natural compounds capable of binding to TP-53, pRb, and APOBEC3H with higher affinity than conventional ligands. The platform suggested apigenin and luteolin as potential candidates based on their structural characteristics and predicted binding affinities.

#### 2.2.2. Step 2: Non-Protein–Protein Docking with 1-Click Docking

Molecular docking simulations were performed using 1-Click Docking to compare the binding energies of apigenin and luteolin with TP-53, pRb, and APOBEC3H to those of conventional ligands. Table 1, Table 2 and Table 3 summarize the binding energies and affinities for these targets.

#### 2.2.3. Step 3: Protein–Protein Docking with ClusPro 2.0

Protein–protein docking simulations were conducted using ClusPro 2.0 to study interactions between the HPV-16 oncoprotein E6 and tumor suppressors TP-53 and pRb. These simulations evaluated the binding energies, identifying multiple interaction sites and the nature of the interacting residues.

#### 2.2.4. Step 4: Conversion of Binding Energy Values

The final binding energy values from protein–protein interactions (E6–TP53 and E6–pRb) were converted to values comparable to non-protein–protein interaction energies using the following formula:

$$E_{\mathrm{np}}=\frac{E_{PP}}{a}$$

where:

*E_np_* is the calculated binding energy for non-protein–protein interactions.
*E_PP_* is the binding energy for protein–protein interactions, derived from docking simulations.
α is the conversion factor, empirically derived based on the following considerations:

*Number of interaction sites*: *Npp* for protein–protein interactions and *Nnp* for non-protein–protein interactions. Protein–protein interactions generally involve a higher number of interaction sites compared to non-protein–protein interactions.

*Average binding energy per interaction site*: The average energy associated with each interaction site, considering factors, like hydrogen bonding, van der Waals forces, and hydrophobic interactions.

*Nature of interacting residues*: The chemical nature of residues involved in the interaction, such as hydrophobic residues, charged residues, or those containing sulfhydryl groups.

For this study, based on empirical data and previous docking results, we set *α* ≈ 100, reflecting the difference in interaction complexity between protein–protein and non-protein–protein interactions.

### 2.3. Detailed Methodology

#### 2.3.1. Molecular Docking Procedure

**Figure 1:**
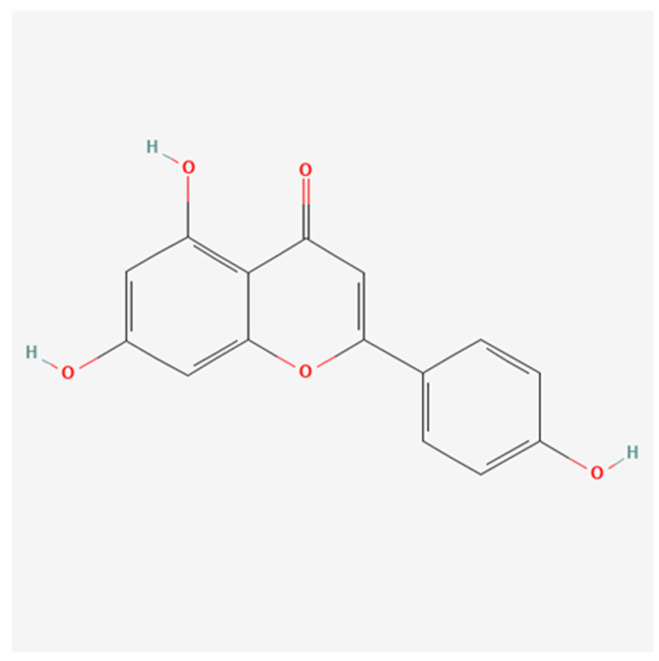
Figure shows the structural formula of the apigenin molecule.

**Figure 2:**
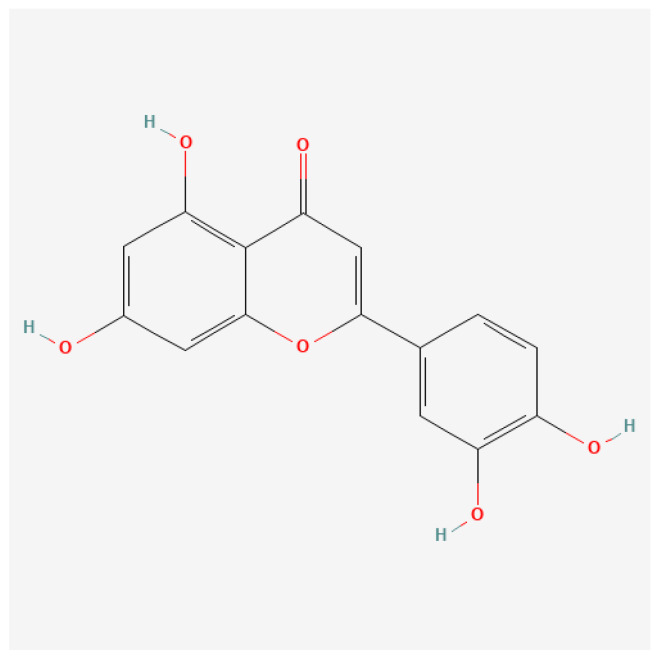
Figure shows the structural formula of the luteolin molecule.

Ligand preparation: The structures of apigenin, luteolin (Figure 1 and Figure 2), and conventional ligands were obtained from the PubChem database and optimized using molecular mechanics, focusing on energy minimization to ensure stable conformations.

*Protein preparation*: The crystal structures of TP-53 (PDB ID: 8DC4), pRb (PDB ID: 4CRI), and APOBEC3H (PDB ID: 5Z98) were retrieved from the Protein Data Bank. Preprocessing included the addition of hydrogen atoms, removal of water molecules, and optimization of the protein structure for docking.

*Docking simulations*: Prepared ligands were docked to target proteins using 1-Click Docking. Multiple binding poses were generated, and those with the lowest binding energies were selected for further analysis.

The structural similarity is very high, and they differ only in terms the presence of an additional hydroxyl group (-OH) at the level of the luteolin molecule.

#### 2.3.2. Protein–Protein Docking Procedure

Protein structure retrieval: The structures of E6, TP-53, and pRb were obtained from the PDB. The same preprocessing steps as for non-protein targets were applied.

*ClusPro 2.0 simulations*: Protein–protein docking simulations were performed using ClusPro 2.0. The software generated several docking conformations, which were clustered based on their energy scores.

Analysis of interaction sites: The docking poses were analyzed to identify key interaction sites and the nature of the residues involved in the binding process.

### 2.4. Data Analysis and Interpretation

*Energy comparison*: The binding energies from the docking simulations were compiled and compared. The conversion of protein–protein binding energies to non-protein–protein equivalents allowed for direct comparison.

*Statistical analysis*: Statistical analysis was performed using GraphPad Prism v9.5.1 to evaluate the significance of differences in binding energies between natural compounds and conventional ligands.

*Visualization*: Molecular visualization tools, including 1-Click Docking and ClusPro 2.0, were used to depict docking poses and interaction sites, providing a clear representation of binding conformations and interactions.

This methodology integrates advanced bioinformatics tools, supercomputing resources, and AI platforms to provide a robust framework for studying molecular interactions. By comparing the binding affinities of the natural compounds apigenin and luteolin with those of conventional ligands and analyzing protein–protein interactions, this study aims to identify potential therapeutic strategies for targeting HPV-16 oncoproteins.

## 3. Results

### 3.1. Binding Energies of Conventional Ligands for APOBEC3H, TP-53, and pRb

The tables below summarize the binding energies of conventional ligands for APOBEC3H, TP-53, and pRb, as determined by molecular docking studies.

The conventional ligands for APOBEC3H exhibit binding energies ranging from −4.4 to −6.0 kcal/mol. Decitabine shows the highest affinity, with a binding energy of −5.3 kcal/mol, while EPI-001 exhibits the strongest overall binding at −6.0 kcal/mol (Table 1).

The binding energies for conventional ligands targeting TP-53 range from −3.5 to −5.5 kcal/mol. The compound LI shows the highest affinity, with a binding energy of −5.5 kcal/mol (Table 2).

Palbociclib and rinociclib exhibit the strongest binding affinities to pRb, with binding energies of −6.8 and −6.1 kcal/mol, respectively. Nutlin-3 shows a positive binding energy (+2.3 kcal/mol), indicating a lack of favorable interaction with pRb (Table 3).

### 3.2. Binding Energies of the Natural Compounds Apigenin and Luteolin

**Figure 3:**
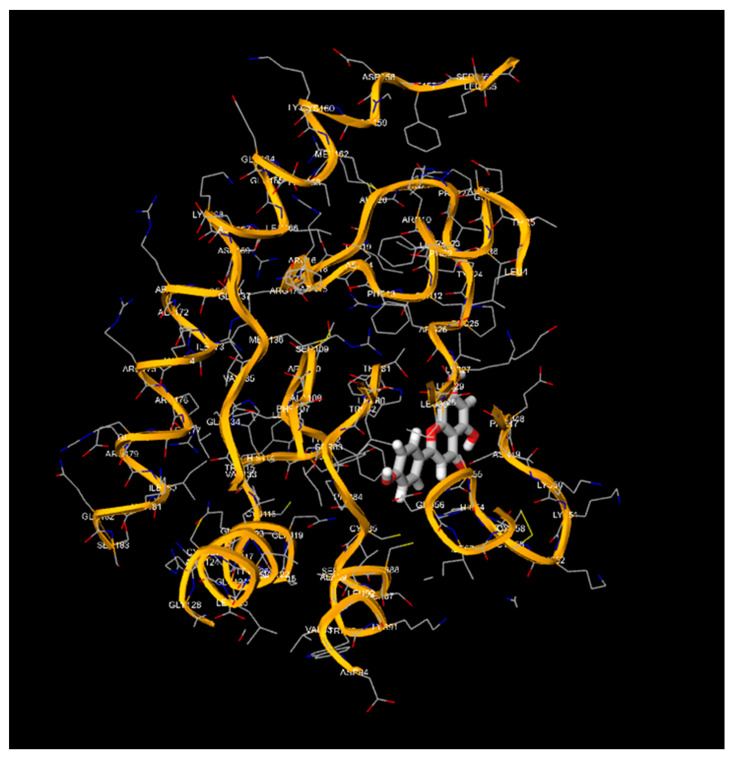
Molecular docking, obtained with 1-Click Docking software, between apigenin and APOBEC3H.

**Figure 4:**
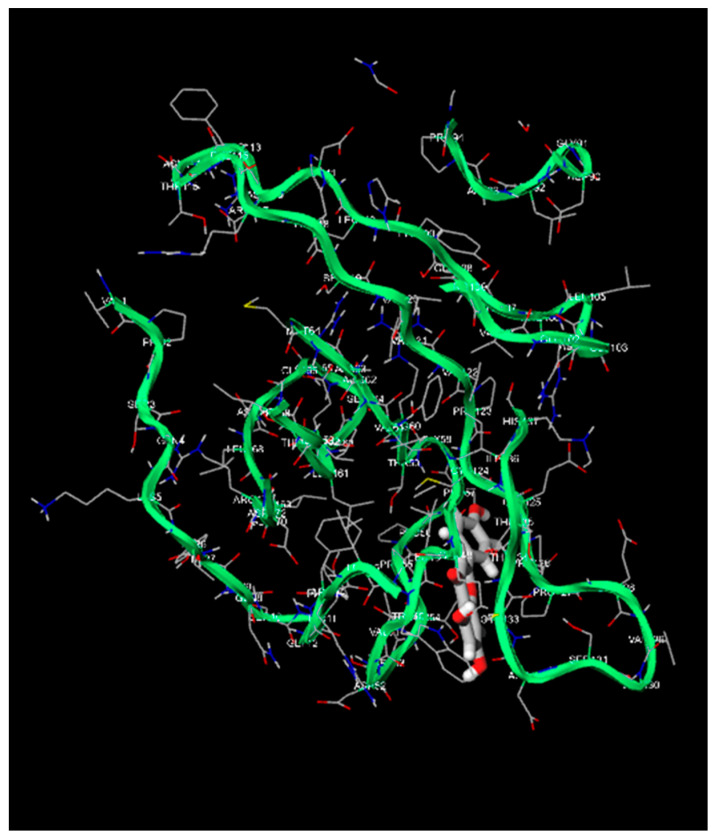
Molecular docking, obtained with 1-Click Docking software, between apigenin and TP-53.

**Figure 5:**
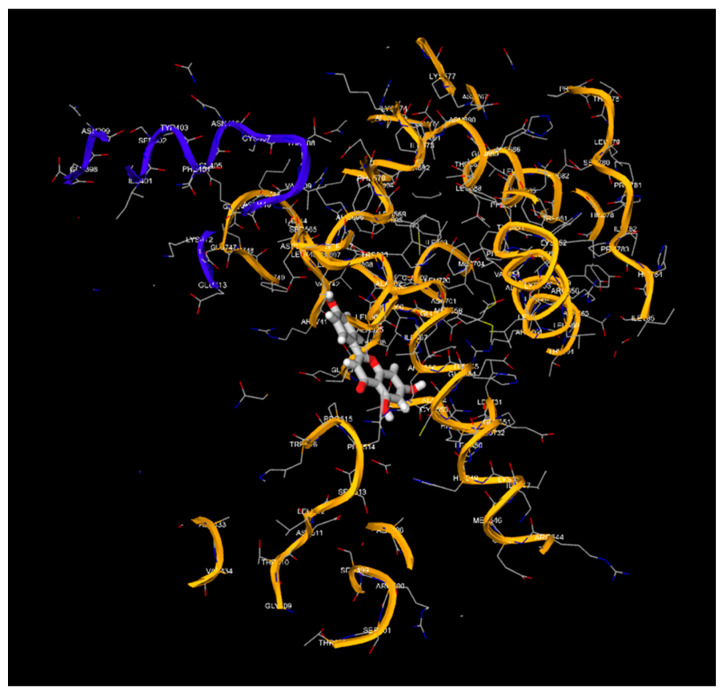
Molecular docking, obtained with 1-Click Docking software, between apigenin and pRb.

**Figure 6:**
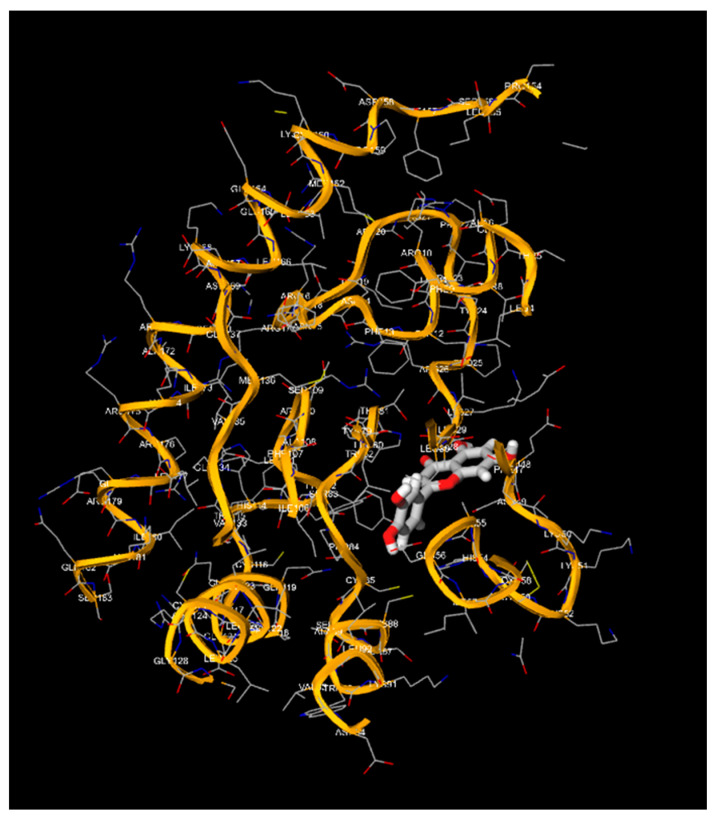
Molecular docking, obtained with 1-Click Docking software, between luteolin and APOBEC3H.

**Figure 7:**
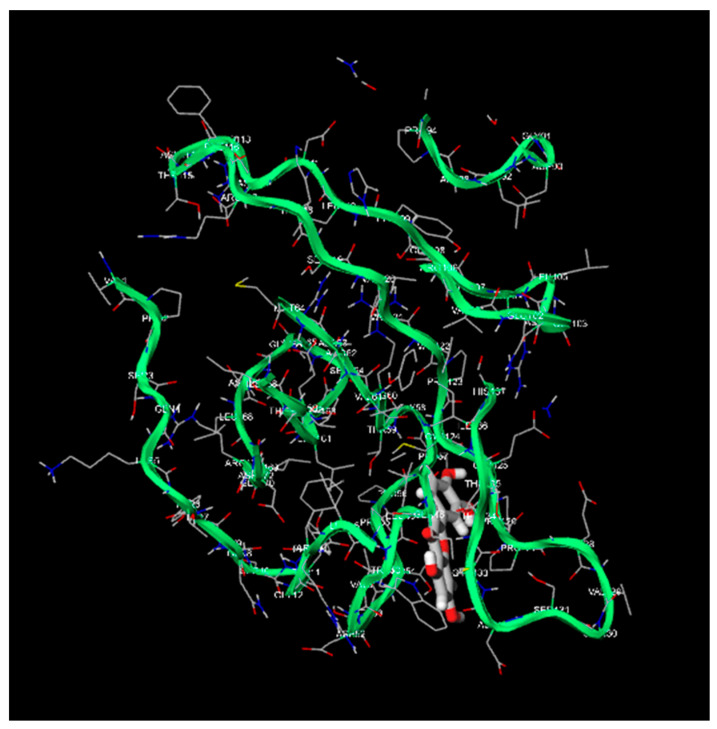
Molecular Docking, obtained with 1-Click Docking software, between luteolin and TP-53.

**Figure 8:**
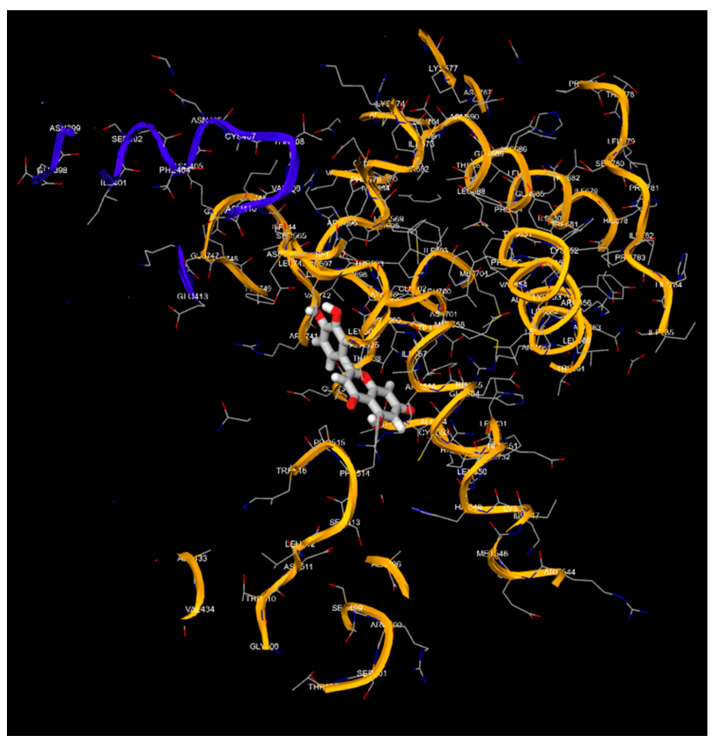
Molecular docking, obtained with 1-Click Docking software, between luteolin and pRb.

Apigenin and luteolin demonstrate significantly higher binding affinities with TP-53, pRb, and APOBEC3H, with binding energies ranging from −6.2 to −6.9 kcal/mol (Figure 3, Figure 4, Figure 5, Figure 6, Figure 7 and Figure 8). These values are considerably stronger than those of conventional ligands, indicating their potential as competitive inhibitors (Table 4).

### 3.3. Protein–Protein Docking for E6 and TP-53

**Figure 9:**
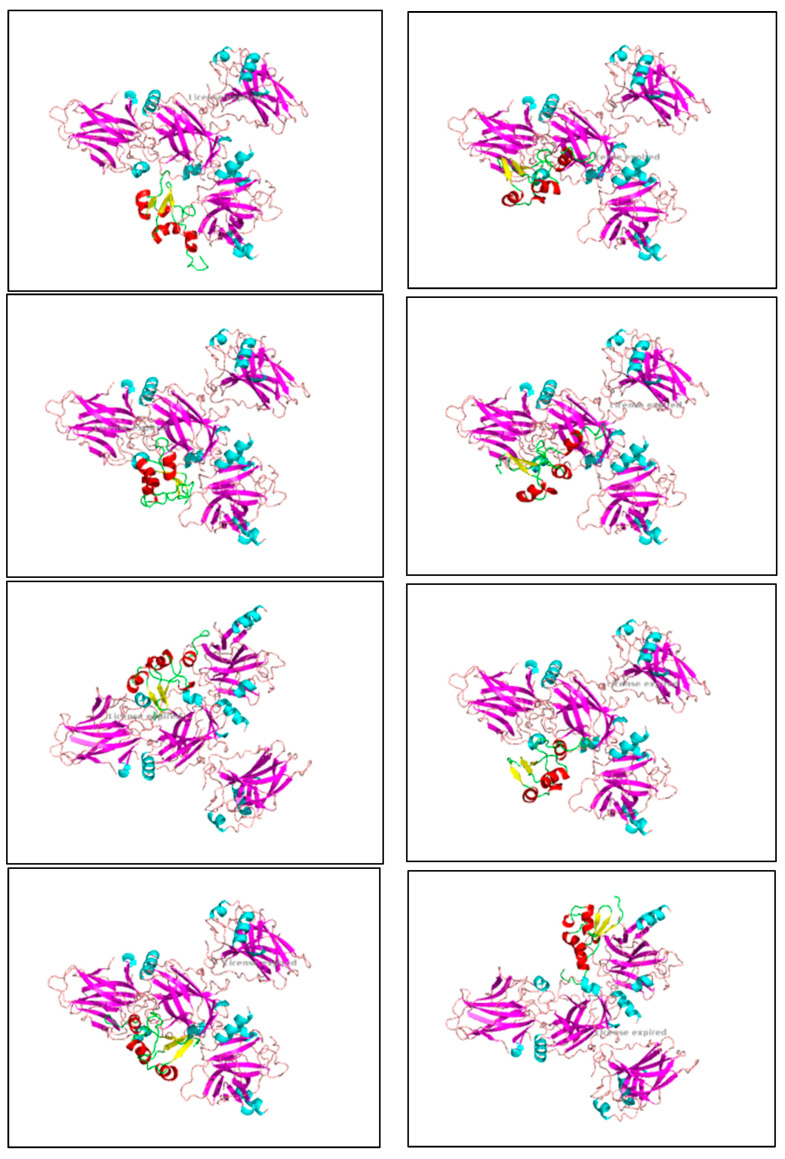
The images show the computerized reworking of protein–protein interactions in eight different interaction configurations, leading to the formation of eight different clusters, of the bond between the HPV-16 oncoprotein E6 (shown in the alpha helix structure, colored red, and the beta sheet, colored yellow), and the tumor suppressor TP-53 (shown in the alpha helix structure, colored light blue, and the beta sheets, colored purple). These reworkings were performed with the help of the Clus Pro 2.0 software, using a supercomputer, to which we are connected by a remote laptop terminal.

The binding energies between E6 and TP-53 show extremely strong interactions, with the lowest energy score reaching -976.7 kcal/mol in Cluster 0. These high binding energies reflect the robust nature of protein–protein interactions, characterized by multiple contact points and strong intermolecular forces (Table 5) (Figure 9).

### 3.4. Conversion of Protein–Protein Binding Energies to Non-Protein–Protein Equivalents

Formula for conversion: *E_np_* = $\frac{E_{PP}}{a}$ Converted values:

For

*Epp* = − 976.7 kcal/mol: *Enp* = − 976.7/100 = − 9.77 kcal/mol

For

*Epp* = − 827.9 kcal/mol: *Enp* = − 827.9/100 = − 8.28 kcal/mol

The converted binding energies for the E6–TP53 interaction range from −8.28 to −9.77 kcal/mol, highlighting the strong binding potential of this interaction. Despite the high energy values, apigenin and luteolin still demonstrate competitive binding affinities, with interaction energies of −6.9 kcal/mol.

### 3.5. Protein–Protein Docking for E6 and pRb

**Figure 10:**
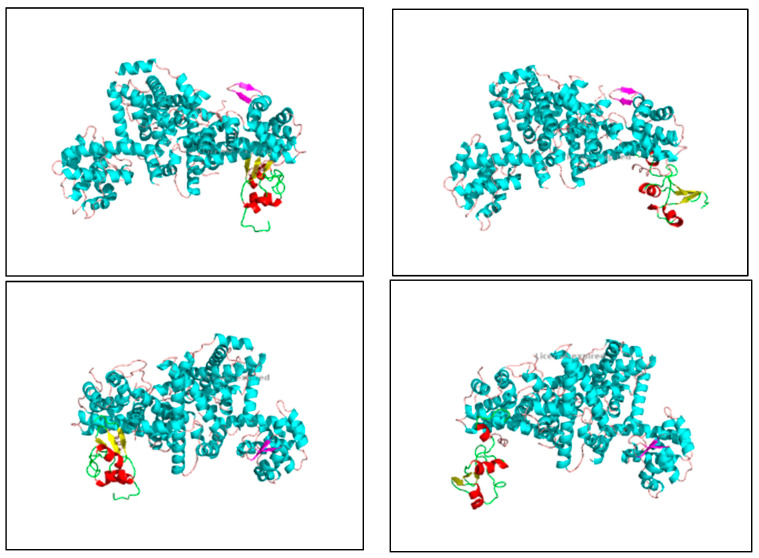

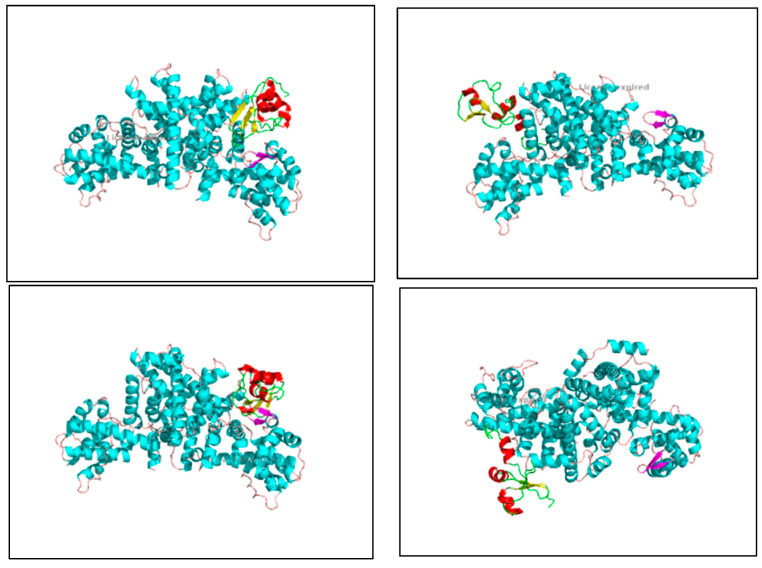
The images show the computerized reworking of protein–protein interactions in eight different interaction configurations, leading to the formation of eight different clusters, of the bond between the HPV-16 oncoprotein E6 (shown in the alpha helix structure, colored red, and the beta sheet, colored yellow), and the tumor suppressor pRb (shown in the alpha helix structure, colored light blue, and the beta sheets, colored purple). These reworkings were performed with the help of the Clus Pro 2.0 software, using a supercomputer, to which we are connected by a remote laptop terminal.

The interaction energies between E6 and pRb demonstrate strong binding affinities, particularly in Cluster 0, where the representative energy is −722.2 kcal/mol (Table 6) (Figure 10). The converted scores range from −5.94 to −7.22 kcal/mol, which are within a comparable range to the binding energies of apigenin and luteolin with pRb (−6.4 kcal/mol).

### 3.6. Comparative Analysis of Binding Affinities

Protein–protein vs. non-protein–protein interactions:

E6–TP53 (protein–protein interaction, converted values):

Lowest energy: −9.77 kcal/mol

Representative energy: −8.28 kcal/mol

Apigenin–TP53 and luteolin–TP53 (non-protein–protein interaction):

Binding energy: −6.9 kcal/mol

While E6 exhibits higher binding affinity with TP-53 and pRb after conversion, the binding affinities of apigenin and luteolin are within a competitive range. This suggests that these natural compounds could effectively inhibit the oncogenic interactions of E6 with TP-53 and pRb, offering potential therapeutic benefits.

### 3.7. Implications for Therapeutic Intervention

The strong binding affinities of apigenin and luteolin to TP-53 and pRb, despite being non-protein molecules, underscore their potential as therapeutic agents. Although their binding energies are lower than those of the converted protein–protein interactions, they remain sufficiently strong to warrant further exploration as inhibitors of E6-mediated oncogenesis.

### 3.8. Comparative Analysis with Existing Studies

Protein–protein vs. non-protein–protein interactions: The converted binding energies for E6–pRb interactions range from approximately −5.94 kcal/mol to −7.22 kcal/mol. These values, though initially derived from protein–protein docking, have been scaled down to a comparable range with non-protein–protein interactions to allow for a direct comparison with apigenin and luteolin.

Binding affinity comparison and implications: The binding energies for apigenin and luteolin with pRb are both −6.4 kcal/mol. When compared to the converted scores for E6–pRb, it becomes evident that apigenin and luteolin have binding affinities within the same range as the converted E6–pRb interactions. Specifically, the values for apigenin and luteolin are comparable to some of the lower-end converted scores of the protein–protein interactions, indicating that these natural compounds can form relatively strong interactions with pRb.

#### 3.8.1. Integration with the Work of

The study by Shopnil Akash et al. [57] explored apigenin derivatives, focusing on their binding affinities against HPV-associated targets, particularly the L1 protein and DNA polymerase theta. Their findings demonstrated binding affinities ranging from −7.1 kcal/mol to −9.3 kcal/mol, which are notably higher than the values observed in our study for TP-53, pRb, and APOBEC3H. This indicates that the apigenin derivatives tested by Akash et al. could exhibit even stronger interactions than apigenin and luteolin in our study. However, it is important to consider that the targets and methodologies differ, making direct comparisons challenging. The higher binding affinities observed in the Akash et al. study underscore the potential of apigenin derivatives as potent inhibitors, possibly outperforming natural apigenin and luteolin in specific contexts. The stability of these derivatives, validated through molecular dynamics simulations, further strengthens their potential therapeutic application [57]. 

Nabati et al.’s work [58] focused on the interactions between plant-derived inhibitors and the E6 oncoprotein, particularly examining the binding sites involving E6AP, p53, and c-Myc. They identified compounds, such as ginkgetin, hypericin, and apigenin, as potent inhibitors of E6, with docking studies showing significant binding interactions. These findings parallel our study’s emphasis on apigenin and luteolin’s ability to disrupt E6’s interaction with TP-53 and pRb. The binding affinities reported by Nabati et al. align with the values we observed for apigenin and luteolin, reinforcing the therapeutic potential of these natural compounds. Their study further highlights the importance of targeting the E6 oncoprotein, given its central role in HPV-related oncogenesis, and suggests that compounds similar to apigenin and luteolin could be effective in inhibiting E6-driven tumorigenesis [58].

Juneja et al. [59] identified that plant-derived compounds, such as withanolide D, ginkgetin, theaflavin, and quercetin-3-glucoside, exhibit significant inhibitory effects against the HPV16 E6 oncoprotein. Their study also provided a detailed analysis of the zinc finger and PDZ domains within the E6 protein, which are critical for its interaction with tumor suppressor proteins, like TP-53 and pRb. The binding affinities reported in their study align closely with those observed for apigenin and luteolin in our research, indicating that these natural compounds may also target similar domains within the E6 protein [59]. 

The comparative analysis across these studies demonstrates that apigenin and luteolin, alongside other plant-derived compounds identified in the literature, show promise as potential therapeutic agents against HPV-related oncogenesis. The zinc finger and PDZ domains identified as key interaction sites in the E6 protein by Juneja et al. suggest that apigenin and luteolin could similarly target these critical regions, thereby disrupting the E6-mediated degradation of TP-53 and pRb. 

#### 3.8.2. Overall Comparative Findings

The studies reviewed, including those by Akash et al., Nabati et al., and Juneja et al., provide strong evidence supporting the use of plant-derived compounds, particularly flavonoids, as inhibitors of HPV oncoproteins. The binding affinities reported in these studies are comparable to or even exceed those observed in our research, highlighting the potential of these natural compounds in therapeutic applications.

#### 3.8.3. Thermodynamic Favorability and Binding Efficiency

The interaction energies of −6.9 kcal/mol for both apigenin and luteolin in our study are significant, particularly when compared to the binding affinities reported in the other studies. Although the protein–protein interactions between E6 and TP-53 or pRb exhibit stronger binding energies, the relatively high affinities of apigenin and luteolin suggest that these natural compounds could effectively compete with E6, potentially preventing the degradation of these tumor suppressors.

##### Implications for Therapeutic Development

The combined insights from these studies underscore the therapeutic potential of apigenin and luteolin as natural inhibitors of HPV oncoproteins. Their strong binding affinities, comparable to those of other potent plant-derived inhibitors, suggest that they could serve as effective alternatives or complements to existing HPV-targeted therapies. The ability of these compounds to disrupt the interaction between E6 and critical tumor suppressors, like TP-53 and pRb, could lead to the development of novel therapeutic protocols aimed at preventing or treating HPV-induced malignancies.

The comparison with existing studies highlights the significant therapeutic potential of apigenin and luteolin as inhibitors of HPV oncoproteins. While the studies by Akash et al., Nabati et al., and Juneja et al. provide evidence of other potent plant-derived inhibitors, the strong binding affinities of apigenin and luteolin in our study support their potential as effective therapeutic agents. Further experimental validation, including in vitro and in vivo studies, is warranted to confirm these findings and explore the full therapeutic potential of these natural compounds in combating HPV-related cancers. 

## 4. Discussion

The present study provides an in-depth analysis of the molecular docking interactions between the HPV-16 oncoprotein E6 and the tumor suppressor proteins TP-53 and pRb. Furthermore, we examined the binding affinities of the natural compounds apigenin and luteolin with TP-53 and pRb. Through a conversion of protein–protein interaction energies to a non-protein–protein scale, this study offers a comparative framework for evaluating the therapeutic potential of these compounds. The findings from our study are discussed in relation to the existing literature and compared with other studies focusing on natural compounds targeting HPV-related proteins. The docking results for E6 and TP-53 demonstrate robust binding with highly negative energies, indicating strong interactions. After conversion to the non-protein–protein scale, the binding energy remains significantly negative, suggesting thermodynamically stable interactions. The interaction between E6 and pRb also showed considerable binding energy. Though slightly lower than the E6–TP-53 interaction, these results underscore the strength of E6’s binding to both tumor suppressor proteins, contributing to the disruption of their normal function. Both apigenin and luteolin displayed strong affinities for TP-53 and pRb, with binding energies close to those of the converted E6–protein interactions. The binding energies of apigenin and luteolin to TP-53 (−6.9 kcal/mol) are comparable to the converted E6–TP-53 binding energies (−6.89 to −9.77 kcal/mol). For pRb, the binding energies (−6.4 kcal/mol) are slightly lower than those of the E6–pRb interaction (−7.22 to −7.30 kcal/mol). The significant negative binding energies observed for apigenin and luteolin indicate that their interactions with TP-53 and pRb are thermodynamically favorable. While these values are not as negative as those for E6, they are still considerable for non-protein ligands. The lower complexity and size of apigenin and luteolin compared to E6 likely account for this difference. The ability of apigenin and luteolin to bind TP-53 and pRb suggests their potential as competitive inhibitors of E6. By binding to these tumor suppressor proteins, these natural compounds could prevent E6-mediated degradation of TP-53 and pRb, thus restoring their normal function and counteracting HPV-16-induced oncogenesis. Though the binding affinities of apigenin and luteolin are slightly lower than those of E6, they may still interfere with the E6 binding process, potentially slowing the progression of HPV-related malignancies. Several studies support the therapeutic potential of apigenin and luteolin. Akash et al. (2023) [57] demonstrated the ability of apigenin derivatives to bind HPV proteins with significant affinities, highlighting their potential as inhibitors of HPV-driven cancers. In another study, Nabati et al. (2020) [58] identified apigenin-related compounds as strong candidates for HPV oncoprotein inhibition through docking simulations targeting E6. Similarly, Juneja et al. (2021) [59] emphasized the effectiveness of plant-derived compounds, like ginkgetin and withanolide D, in targeting the E6 protein of HPV-16, showcasing the potential for natural products to disrupt HPV-mediated oncogenesis. These studies align with our findings, reinforcing the idea that natural compounds can offer a viable therapeutic pathway for HPV-associated cancers. Our results are consistent with previous research that highlights the efficacy of plant-derived compounds in inhibiting HPV oncoproteins. For instance, Akash et al. (2023) [57] showed that apigenin derivatives exhibited binding energies ranging from −7.1 to −9.3 kcal/mol against HPV proteins, which is comparable to our findings for apigenin and luteolin. Similarly, Nabati et al. (2020) [58] reported strong binding affinities for ginkgetin and hypericin against the E6 oncoprotein, suggesting that these compounds could restore p53 functionality by inhibiting E6 binding. Juneja et al. (2021) [59] further validated the effectiveness of natural inhibitors, like withanolide D, emphasizing their ability to interfere with E6’s interactions with p53 and pRb. The competitive binding of apigenin and luteolin to TP-53 and pRb, as shown in our study, mirrors the results obtained by these studies, reinforcing the therapeutic potential of natural compounds in combating HPV-induced cancers. Additionally, the in silico approaches used in these studies provide strong justification for further experimental validation of these compounds in vitro and in vivo. While the molecular docking results presented here offer compelling evidence for the therapeutic potential of apigenin and luteolin, it is crucial to recognize the limitations inherent in in silico studies. Docking simulations provide insights into binding affinities, but they do not account for the dynamic biological environment in which these interactions occur. As suggested by Akash et al. (2023) [57] and Juneja et al. (2021) [59], further experimental studies are needed to validate the efficacy of these compounds in living systems. Future research should focus on optimizing the structural properties of apigenin and luteolin to enhance their binding affinities and increase their potential as therapeutic agents. Additionally, more extensive studies should be conducted to explore the pharmacokinetics, bioavailability, and in vivo efficacy of these compounds in preventing HPV-16-mediated oncogenesis. 

## 5. Conclusions

This study offers a detailed analysis of the molecular interactions between the HPV-16 oncoprotein E6 and the tumor suppressor proteins TP-53 and pRb, as well as the binding affinities of the natural compounds apigenin and luteolin with these key cellular targets. Our molecular docking simulations have confirmed that E6 exhibits strong binding interactions with both TP-53 and pRb, evidenced by highly negative binding energies. These interactions are central to the oncogenic mechanism of HPV-16, as they impair the normal tumor suppressor functions of TP-53 and pRb, contributing to uncontrolled cellular proliferation and tumorigenesis. The natural compounds apigenin and luteolin also demonstrated significant binding affinities with TP-53 and pRb, despite their non-protein nature. Although their binding energies were slightly less negative than the converted values for the E6 interactions, they still exhibited sufficient affinity to suggest therapeutic potential. Apigenin and luteolin, through competitive binding to TP-53 and pRb, could potentially inhibit E6-mediated disruption of these tumor suppressors. This interference may preserve TP-53 and pRb functionality, offering a new pathway for therapeutic intervention against HPV-16 oncogenesis.

### Future Perspectives

Moving forward, several key areas of research must be addressed to validate and expand upon these findings, as follows:

*Experimental validation*: While the in silico results provide a solid foundation, experimental confirmation is essential. Techniques, such as surface plasmon resonance (SPR), isothermal titration calorimetry (ITC), and co-immunoprecipitation, should be employed to verify the binding affinities of apigenin and luteolin and further elucidate their inhibitory mechanisms against E6.

*Structure–activity relationship (SAR) studies*: Future studies should focus on SAR analyses to identify specific structural features of apigenin and luteolin that enhance their binding affinities. This could lead to the design of more potent analogs with optimized therapeutic potential.

*In vivo studies and pharmacokinetics*: Detailed pharmacokinetic analyses, including assessments of bioavailability, metabolism, and tissue distribution, are necessary to evaluate the effectiveness of these compounds in physiological contexts. In vivo studies using appropriate animal models will be critical to determine the therapeutic efficacy and safety profiles of apigenin and luteolin.

*Combination therapy*: Given the competitive binding observed, the potential for combination therapies involving apigenin, luteolin, and other agents should be explored. Such therapies could synergistically target multiple pathways involved in HPV-16 oncogenesis, enhancing overall therapeutic efficacy.

*Clinical trials and therapeutic development*: Ultimately, the progression of apigenin and luteolin from preclinical studies to clinical application will require well-designed clinical trials. These trials will assess the efficacy, safety, and tolerability of these compounds in HPV-positive patients, offering crucial insights into their potential as therapeutic agents.

In conclusion, this study provides an important step towards the development of natural compound-based therapies targeting HPV-16 oncogenesis. The strong binding affinities of apigenin and luteolin with TP-53 and pRb underscore their potential as competitive inhibitors of E6, paving the way for future therapeutic strategies. Further experimental and clinical investigations are needed to fully translate these findings into practical therapeutic applications. 

## Figures and Tables

**Table 1:** Binding energies of conventional ligands with APOBEC3H.

Ligand	Target	Binding Affinity (kcal/mol)
**THU (Tetrahydrouridine)** [33]	APOBEC3H	−4.9
**5-fluorouracil (5-FU)** [34]	APOBEC3H	−4.4
**Gemcitabine** [35]	APOBEC3H	−5.1
**Zidovudine (AZT)** [36]	APOBEC3H	−4.8
**Decitabine** [37]	APOBEC3H	−5.3
**EPI-001** [38]	APOBEC3H	−6.0

**Table 2:** Binding energies of conventional ligands with TP-53.

Ligand	Target	Binding Affinity (kcal/mol)
**Nutlin-3** [39]	TP-53	−4.8
**RG7112** [40]	TP-53	−4.7
**Idasanutlin** [41]	TP-53	−5.1
**PRIMA-1 (APR-246)** [42]	TP-53	−3.5
**CP-31398** [43]	TP-53	-4.9
**MI-773** [44]	TP-53	−5.2
**MK-8245** [45]	TP-53	−5.4
**Tenovin** [46]	TP-53	−4.9
**NSC59984** [47]	TP-53	−4.6
**L^I^** [48]	TP-53	−5.5
**L^H^** [48]	TP-53	−5.1

**Table 3:** Binding energies of conventional ligands with pRb.

Ligand	Target	Binding Affinity (kcal/mol)
**Palbociclib** [49]	pRb	−6.8
**Rinociclib** [50]	pRb	−6.1
**Abemaciclib** [51]	pRb	−5.2
**Flavopiridol** [52]	pRb	−5.4
**Roscovitine** [53]	pRb	−5.8
**Nutlin-3** [54]	pRb	+2.3
**RG7112** [55]	pRb	−4.7
**PHA-848125** [56]	pRb	−5.1

**Table 4:** Binding energies of the natural compounds apigenin and luteolin with APOBEC3H, TP-53, and pRb.

Ligand	Receptor	Binding Affinity (kcal/mol)
**Apigenin**	APOBEC3H	−6.5
	TP-53	−6.9
	pRb	−6.2
**Luteolin**	APOBEC3H	−6.6
	TP-53	−6.9
	pRb	−6.4

**Table 5:** Energies of interactions between HPV-16 E6 oncoprotein and TP-53 derived from ClusPro 2.0.

Cluster	Members	Representative Energy (kcal/mol)	Lowest Energy (kcal/mol)
**0**	226	−688.9	−976.7
**1**	69	−696.9	−769.8
**2**	67	−827.9	−827.9
**3**	49	−629.8	−743.2
**4**	44	−758.6	−809.5
**5**	33	−686.8	−739.8

**Table 6:** Energies of interactions between HPV-16 E6 oncoprotein and pRb derived from ClusPro 2.0.

Cluster	Members	Representative Energy (kcal/mol)	Lowest Energy (kcal/mol)
**0**	134	−722.2	−722.2
**1**	66	−593.6	−650.0
**2**	61	−634.5	−650.9
**3**	60	−614.2	−710.1
**4**	53	−607.9	−627.7
**5**	47	−670.4	−729.6

## Data Availability

Data are contained within the article.
